# Anthropomorphic breast phantoms for quality assurance and dose verification

**DOI:** 10.1120/jacmp.v5i1.1992

**Published:** 2004-05-25

**Authors:** José A. Bencomo, Connel Chu, Victor M. Tello, Sang Hyun Cho, Geoffrey S. Ibbott

**Affiliations:** ^1^ Department of Radiation Physics The University of Texas M. D. Anderson Cancer Center 1515 Holcombe Blvd. Houston Texas 77030; ^2^ Present address: Radiation Therapy Department LDS Hospital 8^th^ & C St. Salt Lake City UT 84143; ^3^ Present address: Medical Physics‐South Florida Region 21^st^ Century Oncology Inc. 2101 Reverside Dr. Ste 101 Coral Springs Florida 33071

**Keywords:** dose calculation algorithms, anthropomorphic phantom, breast cancer treatment

## Abstract

An evaluation of two anthropomorphic breast phantoms, which have been designed for quality assurance and dose verification of radiotherapy treatment of breast cancer patients, is presented. These phantoms are identical in terms of their dimensions and shape and composed of several layers of either Plastic Water™ or tissue‐equivalent material. Both water‐and tissue‐equivalent phantoms include lung‐ and rib‐equivalent components. The phantoms simulate large, medium and small breasts. The value of the phantoms as breast treatment quality assurance tools was assessed by dose measurements with ionization chamber and thermoluminescence dosimeters (TLD) at different points inside the phantom. Measurements were made by irradiating the phantoms under conditions representing the different treatment techniques found by the Radiological Physics Center (RPC) during its dosimetry quality audits. Most irradiations were performed with the water‐equivalent breast phantom. One experiment was performed under consistent irradiation conditions to compare the tissue‐equivalent phantom with the water‐equivalent phantom. Measurements were compared with the dose estimated by the RPC's manual calculations used to check clinical charts of patients entered in a National Surgical Adjuvant Breast and Bowel Project (NSABP) protocol. Measurements were also compared with isodose distributions generated by a commercial radiation treatment planning (RTP) system. In the homogeneous three‐dimensional (3‐D) phantom, fairly good agreement (within 5%) was observed at the NSABP dose prescription point between measurements and 2‐D dose estimation by manual calculations. At the same dose prescription point, but located in the heterogeneous 3‐D phantom, agreement between measurements and a 3‐D RTP system was within about 3%. Manual calculation resulted in overestimation of up to 6%. The general agreement between the TLD measurements and the 2‐D RTP values was within 3% at various off‐axis points, with the exception of a few points far off‐axis, near the high‐dose gradient region at the surface of the phantom.

PACS number(s): 87.53Dq, 87.66Xa, 87.53Xd

## I. INTRODUCTION

Radiotherapy of breast cancer is difficult because of the complex geometry of the target volume, which includes the breast, the adjacent lymph nodes, and the presence of critical organs such as the lungs and heart.^1^ Ideally, the dose distribution should be as homogeneous as possible to avoid areas of under‐dose or overdose, which can result in insufficient tumor control or late sequelae (unacceptable fibrosis) and poor cosmesis.^2^ The critical parameters to verify treatment plans for breast radiotherapy are the accuracy of the dose calculation, the homogeneity of the dose distribution as represented by the plan, and the inclusion of correction for inhomogeneities such as the lung and ribs.

Although several anthropomorphic phantoms have been described in the literature,^3^ ^–^ ^6^ different tissue‐substitute materials^7^ were used in this study to design two prototypic heterogeneous breast‐shaped phantoms. The phantoms are designed to be portable and to allow quick and reliable intercomparison between hand calculations and treatment planning computer calculations. One of the phantoms was water‐equivalent. The other was tissue‐equivalent, and was made of several pieces that simulated breast, lung‐ and rib‐equivalent components, in terms of their radiation absorption properties in the therapy range of photon energies. The phantoms can be used to simulate several different breast sizes and can allow dose homogeneity measurements using TLD, GAF‐Chromic film, or both.

The Radiological Physics Center (RPC) provides quality assurance (QA) support to the cooperative clinical trials sponsored by the National Cancer Institute (NCI). The RPC's QA program for breast radiotherapy protocols includes verification of the dose delivered to the National Surgical Adjuvant Breast and Bowel Project (NSABP) prescription point. Therefore, these phantoms will enable the RPC to verify particular treatment plans and the institution's simulation, dosimetry, and treatment processes.

The phantoms were evaluated under different planning and treatment conditions to validate their usefulness as a QA tool. Comparisons between the calculated dose to the protocol prescription point and dose measurements with and without lung and bone tissues were made. A comparison with an isodose distribution from a treatment‐planning system incorporating inhomogeneity corrections is also presented.

## II. MATERIAL AND METHODS

### Manual calculations of dose delivered to the NSABP prescription point

**Figure 1:**
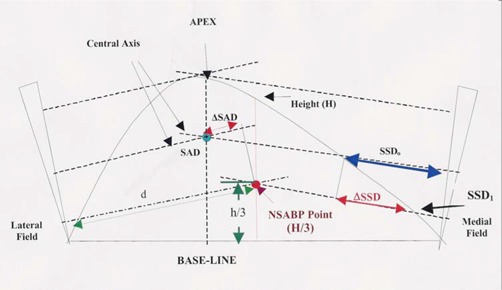
Various geometrical parameters used in the calculation of dose to the NSABP prescription point.

The NSABP protocol prescription point is located at 1/3 the height of the breast's mediolateral plane projection, measured at the breast's height along the baseline's bisector (Fig. 1). This height (the width of the field) multiplied by the length of the treatment fields is a reasonable approximation of the effective field size for the medial and lateral fields. The NSABP prescription point depths are measured along the fan lines for each field. The off‐axis distance to the NSABP prescription point is measured perpendicular to the central ray.

The calculation formulae are shown below. The dose in cGy to the NSABP prescription point for the lateral and medial fields is calculated by the following equations:

For SSD setup: Eq. 1

$$Dose_{NSABP}^{SSD}(cGy)=(MU+\alpha )•OP_{eff}•ISQ(SSD)•DD(FS_{eff},d)•F•WF(FS,d)•TF•OAF_{wedge}$$

Where

(1.a) $${\text{OP}}_{\text{eff}}=\frac{D(d_{\text{max}},\text{Coll})•\text{PSF}({\text{FS}}_{\text{eff}})}{\text{PSF}(\text{collimator})}$$

(1.b) $$ISQ(SSD)={\left[\frac{\left(SSD_{O}+d_{\text{max}}\right)}{\left(SSD_{O}+\Delta SSD+d_{\text{max}}\right)}\right]}^{2}\;,\;\text{and}$$

(1.c) $$F={\left[\frac{\left(SSD_{0}+d\right)•\left(SSD_{1}+d_{\max}\right)}{\left(SDD_{O}+d_{\max}\right)•\left(SSD_{1}+d\right)}\right]}^{2}$$

For SAD setup: Eq. 2

Where

$$Dose_{NSABP}^{SAD}(cGY)=(MU+\alpha )•OP_{eff}•ISQ(SAD)•TMR(FS_{eff},d)•WF(FS,d)•TF•OAF_{wedge}$$

Where

(2.a) $$ISQ(SAD)={\left[\frac{\left(SAD\right)}{\left(SAD+\Delta SSD\right)}\right]}^{2}$$

The parameters used in the equations above are:

*MU*: Number of monitor units used
α: Monitor end effect, a negligible value for modern linear accelerators
$OP_{eff}$: Output factors for the effective field size in cGy/MU at $d_{\max}$ at ${\text{SSD}}_{0}$
*ISQ (SSD*): Inverse square correction due to the difference between the SSD at central axis and the distance to the breast surface at the point of entrance of a ray that passes through the NSABP prescription point for the SSD setup; for the SAD setup, ISQ (SAD) is the correction due to the difference between the SAD distance and the NSABP projected position on the central axis
*F*: Mayneord factor that converts the percentage depth dose (PDD) at nominal ${\text{SSD}}_{0}$ to another ${\text{SSD}}_{1}$
$d_{\max}$ : Depth of maximum dose
$TMR(FS_{eff},d)$: Tissue maximum ratio for the effective field size at the depth of the NSABP prescription point for the SAD setup
$DD(FS_{eff},d)$: Fractional depth dose for the effective field size at the depth of the NSABP point for the
SSD setup
*WF (FS, d*): Wedge factor; function of field size and depth
*TF* is the tray factor
$OAF_{\text{wedge}}$: A correction due to the off‐axis position of the NSABP prescription point with respect to the central axis and the wedge axis; a function of field size and depth

### Phantom Design

**Figure 2:**
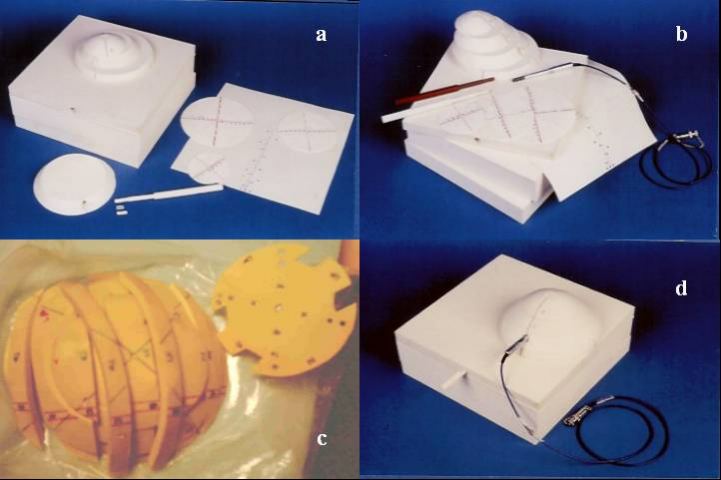
Plastic Water™ phantom with attachments: a) TLD insert, and slabs to simulate small, medium, and large breast sizes. b) Water‐equivalent sheets to hold TLD, ionization chamber insert, c) Water‐equivalent lung insert with cavities to hold TLD, and d) fully assembled phantom with ionization chamber in place.

**Figure 3:**
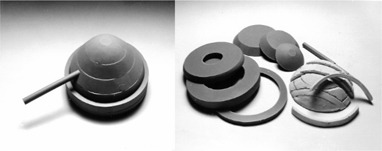
Assembled tissue‐equivalent phantom and its components, including lung and ribs.

**Figure 4:**
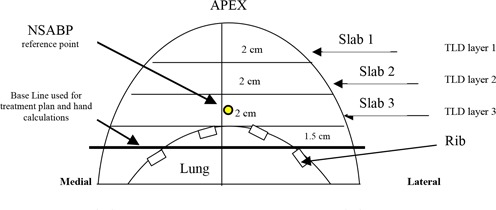
Diagram of breast phantom design (midline slice of large‐size phantom)

A water‐equivalent phantom (Fig. 2) was designed using Plastic Water™ from Computerized Imaging Reference Systems, Inc. (CIRS, Norfolk, VA). Plastic Water™ was used because of its flexibility for making thin slabs and different shapes easily. Cavities were made to accommodate an ionization chamber,

TLD, and GAF‐Chromic film. The material used for the tissue‐equivalent phantom (Fig. 3), also built by CIRS, Inc., was formulated as follows: breast‐tissue substitute (50% glandular and 50% adipose) with a physical density of 0.987; rib tissue‐substitute for bone (50% cortical and 50% spongy) with a physical density of 1.481; and lung‐tissue substitute with a physical density of 0.24. Both water‐ and tissue‐equivalent phantoms consisted of six different 2cm thick slabs and included four 1‐mm thick layers to hold the TLD. A water‐equivalent insert identical in shape to the lung‐equivalent insert was built to complete the homogeneous water‐equivalent phantom. The lung and rib inserts of these two phantoms could be interchanged for various applications. The cylindrical cavity for the ionization chamber lies at the NSABP protocol prescription point for the full‐size phantoms. A conical shape was chosen for the breast phantoms not only for easy assessment of measured results but also to facilitate beam setup and geometrical alignment during simulation and treatment. The size of the fully assembled breast phantoms was based on the breast size of a representative large‐breasted patient as determined from a review of the NSABP study charts. Three different breast sizes (large, 6 cm; medium, 4 cm; small, 2 cm) were simulated by removing 0, 1, and 2 top slabs from the large phantoms, respectively, as seen in Fig. 4. Note that the breast apex shape was not preserved, but was similar to a resected breast.

The location of the NSABP prescription point for each phantom size simulated depends, not only on how many slabs are used (breast size), but also on the position of the baseline used in the treatment plan simulation and hand calculations. The location of the NSABP prescription point for the medium and small phantom sizes was obtained by changing the position of the baseline used for the large‐size phantoms. In this report, point dose measurements in the three phantom sizes were performed at the corresponding NSABP reference points, for each phantom size.

### Treatment Simulation

**Figure 5:**
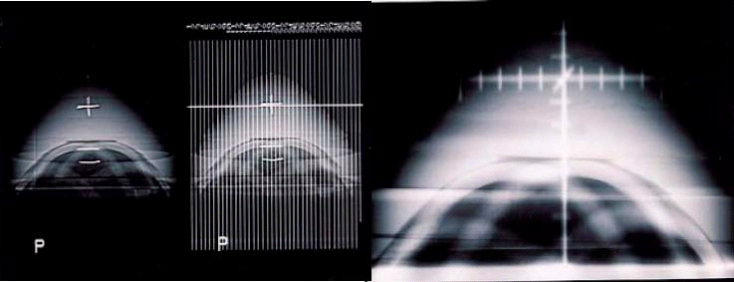
a) CT images of the inhomogeneous water‐equivalent phantom at the midline slice (5‐mm slice thickness); b) Scout view, and c) Simulation film of the tangential treatment setup.

Computed tomography (CT) images of the inhomogeneous water‐equivalent phantom, including the ribs and lung inserts (Fig. 2), were obtained from a CT scanner (General Electric, Milwaukee, WI) using contiguous slices of 5‐mm thickness. Figs. 5a and 5b show the phantom midline CT slice and the CT scout view, respectively. The midline slice contour was used for two dimensional (2‐D) treatment planning and hand calculations. The breast phantom was also simulated on a Simulix‐Y simulator (Oldelft, Fairfax, VA). This simulation was performed to evaluate the capability of the phantom to provide reasonable information for treatment planning. The breast contour, measured from the CT scans and the isocenter was marked on the phantom. Simulation films were obtained from the tangential treatment setup Fig. 5(c).

### Breast phantom treatment

Breast treatment deliveries at different institutions monitored by the RPC cover a large range of techniques. Hence, the breast phantom was irradiated with several common tangential breast‐treatment techniques: parallel opposed offset fields, and co‐planar oblique fields with and without a wedge. The offset fields were defined either by asymmetric jaws or by half‐beam block devices. Some irradiation conditions investigated in this study might not have resulted in a clinically acceptable dose distribution, due to field combinations and/or wedge angles chosen for a symmetrically shaped breast phantom. However, they were considered appropriate for the purpose of evaluating the RPC manual calculation and radiation treatment planning (RTP) systems. Most irradiations were performed with the water‐equivalent breast phantom. One experiment was performed under consistent irradiation conditions to compare the tissue‐equivalent phantom with the water‐equivalent phantom. In detail, dose measurements at the NSABP point were made to compare the water‐equivalent phantom with the tissue‐equivalent phantom. The difference in dose between the phantoms was within 1% of each other as theoretically expected (i.e., $\left[{\left(\mu /\rho \right)}_{\text{muscle}}/[{\left(\mu /\rho \right)}_{\text{water}}=0.99\right)$.

To reduce the amount of effort, several irradiation field combinations were evaluated and compared using an ionization chamber rather than using a large number of TLD measurements. The photon beams used were Cobalt‐60, and the 6 MV photon beams from several linear accelerators: Clinac 2100, Clinac 6/100, and Clinac 1800 (Varian Oncology Systems, Palo Alto, CA). However, only the results from Cobalt‐60 and one of the 6 MV photon beams will be presented because the results were similar for the 6 MV photon beams.

### Treatment‐Planning Calculations

The RTP systems used in this study were Target II (General Electric) and RenderPlan (Elekta Oncology Systems, Inc, Norcross, GA). Both RTP systems used patient data, treatment‐machine data, and setup information such as the position of the beams and beam modifiers with respect to the patient.

**Figure 6:**
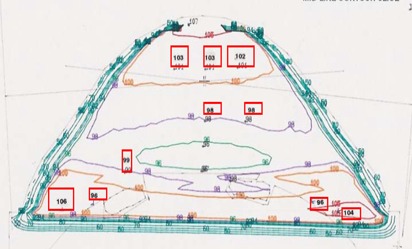
Comparison of TLD measurements with isodose plan not corrected for inhomogeneities.

**Figure 7:**
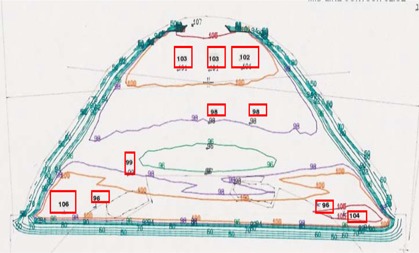
Comparison of TLD measurements with isodose plan corrected for inhomogeneities.

The midline CT scan contour of the homogeneous water‐equivalent phantom was used for 2‐D dose calculations, comparing only the dose at the NSABP prescription point. Target II RTP system performed conventional 2‐D dose calculations; the CT images of the phantom were used by the Target II RTP system to generate isodose curves for the phantom treatment with and without inhomogeneity corrections (see Figs. 6 and 7). The Target II inhomogeneity correction algorithm was based on a semi‐empirical equivalent tissue air ratio (ETAR) designed specifically for CT‐pixel‐based calculations.^8^

CT images of the inhomogeneous water‐equivalent phantom were utilized for 3‐D dose calculations. RenderPlan used a modified Clarkson sector integration algorithm (Irreg. Program) to simulate the 3‐D dose distribution inside a patient.

### Dosimetry

The output calibration of the treatment machines was performed using the AAPM TG‐21 protocol^9^ in a standard 30 cm×30 cm×30−cm solid Plastic Water™ phantom. A Farmer‐type ionization chamber was used for measuring beam outputs. Tello et al.^10^ have investigated the water‐equivalence of water‐equivalent plastics for dose evaluations in the range of photon energies used for breast treatment (i.e., Cobalt‐60 to 6‐MV photon beams and for 4‐ to 25‐MeV electron beams) and found good agreement. A Farmer‐type ionization chamber was also used for absolute dose measurement inside the breast phantom. The ionization chamber measurements became the reference for all other relative measurements using TLDs. TLD‐100; LiF chips (3 mm×3 mm×1 mm) were used for relative measurements. The TLDs were irradiated with their largest surface area parallel to the beam axis. Two TLD chips were placed inside a water‐equivalent plug with the shape of the ionization chamber. The plug was then placed at the NSABP prescription point to obtain the TLD calibration.

## III. RESULTS

### Breast‐size effects

Tables I and II show the effect of breast phantom size on the dose to the NSABP prescription point under the condition of open and wedge field combination. Ion chamber measurement results are presented here for the contribution of each individual irradiation field. The doses to the NSABP point in the medium‐ and small‐size breast phantoms were measured at the chamber position in the slab cavity corresponding to the large‐breast phantom (see Fig. 4). The results from the hand calculation for open fields always overestimated the dose to the NSABP point for the Cobalt‐60 treatment. On the other hand, the RPC's hand calculations consistently underestimated the dose for both open and wedged 6 MV photon beams, in comparison with ion chamber measurements.

**Table I:** Breast size effect on the dose at the NSABP prescription point using the homogeneous water‐equivalent phantom; Cobalt‐60 treatment

Field Set Up 9 cm(w)×19 cm(L) 80 cm SAD	Phantom Size	Ion Chamber Measurements (cGy/min)	Hand Calculat ion (cGy/min)	Calc/Meas	Calc/Meas. (LAT+MED) vs. Breast size
Oblique, open field MEDIAL FIELD (MED)	Large	117.6	123.5	1.05	1.04
	Medium	117.0	120.5	1.03	1.02
	Small	112.3	114.4	1.02	1.00
Oblique, 45° wedged field LATERAL FIELD (LAT)	Large	102.5	104.6	1.02	
	Medium	102.0	102.1	1.00	
	Small	98.8	96.9	0.98	
9 cm(w)×19 cm(L) 82.7 cm SSD	(with half‐beam block breast bridge)				
Open Field MEDIAL FIELD (MED)	Large	92.7	96.4	1.04	1.02
	Medium	92.5	93.2	1.01	1.00
	Small	88.5	90.7	1.02	1.00
45° wedged field LATERAL FIELD (LAT)	Large	61.8	61.7	1.00	
	Medium	61.6	60.3	0.98	
	Small	59.4	57.5	0.97	

**Table II:** Breast size effect on the dose at the NSABP prescription point using the homogeneous water‐equivalent phantom; 6‐MV x‐ray treatment

Field Set Up 9 cm(w)×19 cm(L) 100 cm SAD	Phantom Size	Ion Chamber Measurements (cGy/MU)	Hand Calculation (cGy/MU)	Calc/Meas	Calc/Meas (MED+LAT) vs. Breast size
Oblique, open field MEDIAL FIELD	Large	0.869	0.863	0.99	0.97
	Medium	0.869	0.850	0.98	0.96
	Small	0.843	0.830	0.98	0.97
Oblique, 45° wedged field LATERAL FIELD	Large	0.481	0.459	0.95	
	Medium	0.480	0.448	0.93	
	Small	0.470	0.446	0.95	
9 cm(asym w)×19 cm(L) 100 cm SSD	(with half‐beam block asymmetric jaws)				
Open Field MEDIAL FIELD	Large	0.868	0.863	0.99	0.99
	Medium	0.865	0.854	0.99	0.98
	Small	0.838	0.834	1.00	0.99
45° wedged field LATERAL FIELD	Large	0.389	0.379	0.98	
	Medium	0.387	0.372	0.96	
	Small	0.377	0.370	0.98	

The worst agreement up to 7% between ion chamber measurements and hand calculations was seen for an oblique 45°‐wedged 6 MV beam. The agreement between ion chamber measurements and hand calculations was within 5%, when the total dose to the NSABP prescription point contributed from both medial and lateral fields was considered.

### Effect of lung and ribs on the dose to NSABP prescription point

Treatment plans were generated with and without heterogeneity correction using a commercial RTP system (Render Plan). The phantoms were irradiated under several different conditions, including SSD and SAD setup, with and without the lung/rib material. Several treatment techniques were also used, including oblique field and asymmetric jaws. In Table III, the results of ion chamber measurements at the NSABP prescription point are shown for one of the breast treatment cases studied. The results of the RPC manual calculation for the homogeneous phantom are also shown for comparison. The agreements for the homogeneous phantom case were all within 4%, with the median at approximately 2%. In the cases shown in Table III, the manual calculations agreed equally or better with measurements.

**Table III:** Agreement between ionization chamber measurements by hand calculations and RTP calculations.

Machine Energy (6 MV)	Setup	Field Size (W×L)(cm×cm)	RTP	Breast Phantom	Wedge Angle	RPC Meas.	Hand Calc.	RTP Calc.	Meas./RTP Calc.	Meas./Hand Calc.
						cGy/MU @ NSABP Point				
Clinac	SSD		Render	No Lung	30°	0.438	0.441	0.438	1.00	0.99
1800	Asym	9×16	Plan		45°	0.315	0.328	0.315	1.00	0.96
	Jaws			Lung	30°	0.430	—‐	0.432	1.00	—
					45°	0.307	—‐	0.315	0.98	—

The effect of the presence of the lung and ribs in the photon field appears to reduce the dose contribution to the NSABP dose prescription point by approximately 1 to 2%. At the NSABP dose prescription point, the agreement between the measurements and 3‐D RTP calculations was within ±3%, whereas the manual calculation would result in an overestimation of the dose up to 6%. The NSABP dose prescription point is located 1 cm from the apex of the lung. The amount of lung included in the treatment ranged from 1.3 to 2.2 cm of the breast apical height.

### Comparison of TLD dose measurements with RTP midline isodose distribution

The water‐equivalent phantom was irradiated under identical conditions with and without lung/rib materials. The latter condition was simulated by replacing the bottom two slabs (lung/rib section) by two water‐equivalent slabs of the same dimensions (see Fig. 2c).

Figs. 6 and 7 show the corresponding isodose distributions obtained with the Target II RTP system. Shown in the figure are the contours of the midline CT slice for the large‐size phantom, without and with inhomogeneity correction, respectively.

The isodose distributions in the two figures differ drastically in the region below the NSABP point location. Specifically, Fig. 7 shows hot spots caused by the presence of the lung/rib inhomogeneity.

The numbers inside the boxes in Figs. 6 and 7 represent the relative TLD measurements normalized to the isodose value at the NSABP point. In both cases, the agreement between the TLD measurements and the plan isodose was within 4%. TLD measurements were performed inside the lung region with and without the lung insert. Fig. 7 shows the agreement with the heterogeneity‐corrected plan to be within 4% in that region.

### Comparison of TLD measurements with RTP isodose distribution at off‐axis points

**Figure 8:**
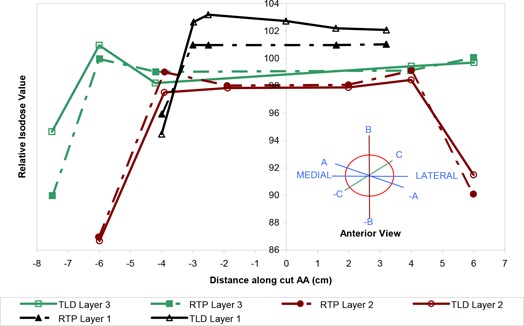
(a) Off‐axis agreement between TLD measurements and RTP isodose values along cut AA (cm)

As seen in Figs. 2 and 4, the TLDs were placed along various radial directions in the circular layers and sandwiched within the breast phantom slabs. The diagram inserted in Fig. 8a describes the three directions (i.e., AA, BB, and CC) of the location of the TLDs in the coronal plane of the large‐size phantom. Each graph 8a, 8b, and 8c shows a comparison between TLD measurements and the isodose values generated by the Target II RTP System along one of the above‐mentioned directions.

The colored curves in each graph show the results for a midline CT slice, as shown in Fig. 5, of the inhomogeneous large‐size phantom. The general agreement between the TLD measurements and the RTP isodose values is within 3% with the exception of a few points far off‐axis, near the high‐dose gradient region at the surface of the phantom.

### Error Analysis

**Figure 9:**
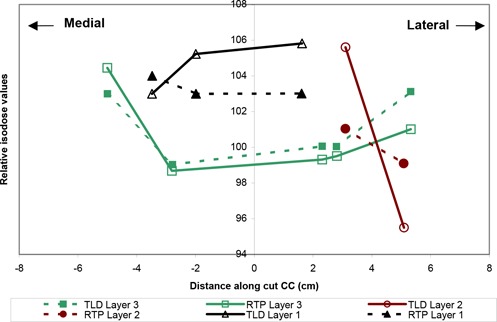
(b) Off‐axis agreement between TLD measurements and RTP isodose values along cut BB (cm).

The TLDs were placed with their 1‐mm thickness orthogonal to the direction of the photon beam. The accuracy of the TLD chips in that direction was assessed by measuring the percentage depth dose along the central axis for a 10 cm×10 cm field size on a Cobalt‐60 beam. Comparison with BJR #17 tables^11^ is shown in Fig. 9. The worst‐case disagreement from the results of two batches of TLD was about 3%. This uncertainty includes three factors: 1) batch sensitivity was within ±2.5%; 2) effective measurement position of the chip was within 1.5 mm (one half of 3 mm chip width); and 3) partial chip shadowing (chip was staggered as a function of depth with maximum overlap of less than 1/5 of the chip's width). The uncertainty in ionization chamber calibration plus the ionization chamber measurements^12^ ^,^ ^13^ was approximately 1.5% and the dose calculation can be estimated from the errors in output factor measurements (1.6%), wedge factor (2%), and percentage dose measurements (1%), tray factor (2%). The total error was approximately 3.4%. The uncertainty in the measured/calculation ratio was approximately 3%.

**Figure 10:**
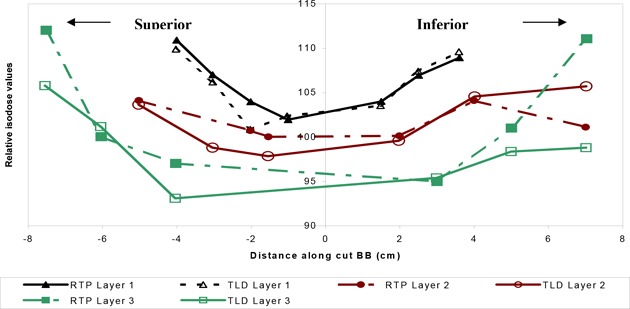
(c) Off‐axis agreement between TLD measurements and RTP isodose values along cuts CC (cm).

## IV. DISCUSSION

The RPC continues developing advanced QA techniques and tools to monitor the quality of treatment of patients entered into NCI clinical trials that use technologically advanced treatment planning and dose‐delivery methods. In this regard, the RPC has developed several anthropomorphic phantoms, including the anthropomorphic breast phantoms presented in this paper. Before these phantoms can be used in the field as QA tools, they need to go through rigorous evaluation. This paper summarizes some of the measurements performed to accept the breast phantoms as reliable QA devices.

As mentioned earlier, some part of the discrepancies between ion chamber measurements and hand calculations could be due to an inaccurate estimation of the photon scatter during the hand calculations. In general, the 2‐D hand calculation tends to overestimate the full‐size breast volume because of the missing phantom material in the third dimension. As the breast size is reduced, therefore, the agreement between hand calculations and measurements for open Cobalt‐60 beams tends to improve. In addition, the backscatter for a Cobalt‐60 beam is larger than that for a 6‐MV photon beam,^8^ which might be a reason why the agreement was generally better for the open 6 MV photon beams. However, these explanations do not appear to be satisfactory, in terms of explaining the somewhat pronounced discrepancies for wedged Cobalt‐60 and 6 MV photon beams. The observed discrepancies for wedged beams might be partially due to a simplified estimate (single slope wedge gradient) of the wedge correction factors in the hand calculations, which does not specifically account for the non‐linear wedge gradient changes across the wedge profile. Furthermore, it has been reported in the literature that oblique incidence of a photon beam on a breast phantom is very sensitive to setup errors.^5^ ^,^ ^6^ These errors could result in large discrepancies in the dose to the reference point. We did not investigate this phenomenon in our studies, and further investigation on this issue may be necessary.

**Figure 11:**
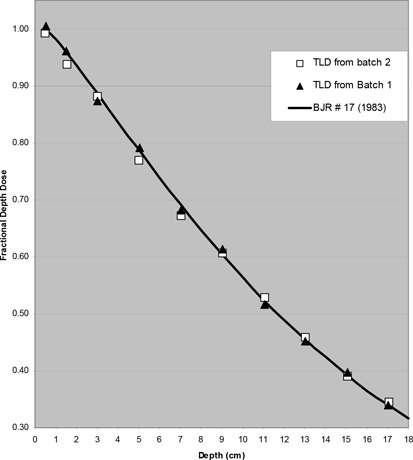
Co60 fractional depth dose (10 cm×10 cm, 80 cm SSD).

As 3‐D treatment planning is routinely performed for the majority of radiation treatments including breast treatment, the verification of 3‐D dose distributions could be an essential part of the QA program. In general, it is very difficult to accurately measure a full 3‐D dose distribution, in spite of recent advances in 3‐D dosimeters such as gel dosimetry. Therefore, as a practical alternative, researchers have developed anthropomorphic phantoms in conjunction with 2‐D dosimeters (e.g., film) or point dosimeters (e.g., TLD) to measure the doses at reference points and a particular plane inside the phantoms. Although this approach has been applied to many treatment sites, little has been reported for breast, especially for breast phantoms that include the lung and other inhomogeneities such as ribs. Accurate dose assessment for breast treatments cannot be achieved without taking into account these inhomogeneous anatomic structures, which are presented in the radiation field. As intensity‐modulated radiation therapy (IMRT) for breast becomes feasible, an accurate dose assessment and verification are essential for successful treatments. Certainly, the phantoms developed in this study could serve as simple and versatile QA tools for the IMRT (or 3‐D conformal RT) of breasts. In particular, they might be used as a credentialing tool during possible multi‐institutional clinical trials for breast 3‐D CRT or IMRT treatments in the future.

## V. CONCLUSIONS

Versatile anthropomorphic breast phantoms have been developed to facilitate the assessment of dosimetry in the breast treatment process. These phantoms are designed to simulate treatments with and without inhomogeneities such as lungs and ribs. These phantoms may serve as a valuable tool for quality assurance of breast treatments by providing a quick assessment of the accuracy of the dose delivery to the breast. At the NSABP dose prescription point located in a homogeneous 3D phantom, a fairly good agreement within 5% was observed between measurements in a 3‐D homogeneous phantom and 2‐D dose estimation by manual calculations. At the same dose prescription point but located in a heterogeneous 3‐D phantom, the agreement between measurements and a 3‐D RTP system's calculated values was within about 3%, whereas the manual calculation resulted in an overestimation of the dose up to 6%. The general agreement between the TLD measurements and the 2‐D RTP values was within 3% at various off‐axis points, with the exception of few points far off‐axis, near the high‐dose gradient region at the surface of the phantom.

## ACKNOWLEDGEMENTS

The anthropomorphic phantoms described in this work were originally designed, developed, and commissioned by Victor M. Tello, MS, while he was at the RPC. We acknowledge the engineers at CIRS Inc, who built the water‐equivalent phantom, the breast‐tissue‐ and bone‐equivalent materials. We would also like to recognize the help and expertise of the following staff of the Department of Radiation Oncology at M. D. Anderson Cancer Center: Eric Strom, MD, Becky Zikes, BS, CMD, Kim Hale, RT(R)(T), Agnes Potovsny, David Lege, RT, CNMT, Peter Balter, PhD., Nathan Wells, MS, Huy Duong, BS, and Mr. Ferrell Munson. We greatly appreciate the secretarial support of Mr. James DeVoge.

This investigation was supported by PHS grant CA 10953 awarded by the National Cancer Institute, DHHS.
